# CMLPS-N1: a novel preclinical cell line model for canine mammary tumor and its application in therapeutic screening

**DOI:** 10.1080/01652176.2026.2614697

**Published:** 2026-01-10

**Authors:** Xiao Wang, Han Zhou, Qian Zhang, Yihan Liu, Lixin He, Wenxuan Li, Bin Ma, Lihong Luo, Lijun Guo, Changwei Qiu

**Affiliations:** a Department of Clinical Veterinary Medicine, College of Veterinary Medicine, Huazhong Agricultural University, Wuhan, China; b School of Physical Education and International Equestrianism, Wuhan Business University, Wuhan, China

**Keywords:** Canine mammary tumor, liposarcoma, canine model, cell line establishment, chemotherapy drug screening, MDM2-positive

## Abstract

Canine mammary tumor is the most common tumor in intact female dogs and poses a growing health burden due to its high malignancy rate and increasing canine populations. However, research on sarcomatous subtypes has been hindered by a lack of representative cell lines. Here, we successfully established a novel canine mammary liposarcoma cell line, designated CMLPS-N1, which represents the first such model derived from a spontaneous tumor. This cell line has been stably maintained for over 80 passages and exhibits an abnormal karyotype, high proliferative and migratory capacity, and strong tumorigenicity in mouse xenografts. Molecular profiling confirmed a phenotype consistent with liposarcoma (MDM2+) and mesenchymal origin (Vimentin+/N-cadherin+), alongside high-risk markers (p53+/Ki67+/Notch1), and hormone receptor expression (ER/PR), while being negative for epithelial (PCK) and HER-2 markers. We used functional assays, including cell proliferation, colony formation, wound healing, and transwell invasion, to confirm its aggressive phenotype. Furthermore, cytotoxicity testing with four chemotherapy agents further supports its utility as a preclinical model for therapeutic screening and mechanistic research. The establishment of CMLPS-N1 enriches the canine mammary tumor cell line repository and provides a valuable experimental model for studying disease mechanisms, developing therapies, and facilitating translational applications.

## 1. Introduction

Canines have become an integral part of human society, serving as working animals in tasks such as visual guidance, search and rescue, and as companion animals providing emotional and psychological benefits. The estimated dog populations reached 94.2 million and 50.85 million in the United States and China, respectively (Finley et al. 2006; Alvarenga et al. 2021), underscoring the significant impact of canine health on public well-being and the economy. Among various health issues affecting dogs, canine mammary tumor (CMT) is particularly concerning due to its high incidence and malignant potential (Vazquez et al. 2023). From 2017 to 2021, CMT accounted for 46.7% of all canine tumor cases, surpassing skin tumor as the most common type (Zheng et al. 2022), and over 50% of these cases were malignant (Ariyarathna et al. 2022). It is imperative to conduct further research and develop effective interventions in this field.

CMT is histogenetically categorized into two principal groups: epithelial-derived and mesenchymal-derived neoplasms (Goldschmidt et al. 2011). Malignant epithelial-derived tumors are conventionally termed mammary carcinomas. Major subtypes include simple carcinomas of a single luminal or myoepithelial cell type; complex carcinomas with a biphasic population of both lineages; ductal carcinomas forming characteristic tubular structures; and special types such as squamous cell and inflammatory carcinoma. Conversely, malignancies arising from mesenchymal tissues are collectively designated as canine mammary sarcomas (CMS) (Tavasoly et al. 2013). Due to the predominance of epithelial-derived mammary tumors in clinical cases, their molecular mechanisms, treatment strategies, and prognostic evaluation have been extensively studied (Zhou C et al. 2023; Wang X et al. 2024; Wu et al. 2024). By contrast, research on CMS remains relatively limited and has not yet been comprehensively investigated.

CMS represents a malignant neoplasm originating from the stromal mesenchymal tissue, characterized by local invasiveness, high metastatic potential and poor prognosis (Turna et al. 2023). Major histological subtypes of CMS include liposarcoma, osteosarcoma, chondrosarcoma, and fibrosarcoma (Hampe and Misdorp 1974). Among these, canine mammary liposarcoma (CMLPS) is a rare subtype resulting from the malignant transformation of lipoblasts within the mammary mesenchymal tissue, and is associated with pronounced malignant potential.

The development of tumor cell lines represents a cornerstone in cancer research due to their indispensable role in modeling disease mechanisms and evaluating therapeutic strategies. However, currently available CMT cell lines remain scarce worldwide, predominantly originating from mammary carcinomas and displaying heterogeneous biological characteristics (Ren et al. 2022; Park et al. 2024). This scarcity is particularly pronounced in the field of CMS, where cellular models are exceptionally limited. To date, only one CMS cell line has been reported: MCO-Y4, a canine mammary osteosarcoma cell line established by Kawabata et al. in Japan (Kawabata et al. 2006). However, to our knowledge, no CMLPS cell line has been reported in the literature. This lack of research materials significantly hinders the exploration of CMS biological behavior, molecular drivers, and potential therapeutic targets.

Therefore, diversifying and expanding this cell line repertoire is essential to advance both fundamental and translational research endeavors. In this study, we established a novel CMLPS cell line, designated CMLPS-N1, and characterized its genetic profile, morphological features, functional properties, and pharmacologic sensitivities, thereby providing a valuable experimental model for future CMT research and helping to alleviate the critical shortage of tools available for studying this aggressive disease.

## 2. Materials and methods

### 2.1. Tumor sample

Tumor tissues were surgically removed from the dogs suffered from mammary tumors in a veterinary hospital, following standard operation procedures and with informed owner approval. Part of the tissues were immediately collected to isolate primary tumor cells, and the other part was fixed in 4% formaldehyde solution for further pathological diagnosis by histology and immunohistochemistry (IHC) to confirm the characteristic and classification of tumors according to Goldschmid (Goldschmidt et al. 2011). Primary cultures from 30 distinct female dogs were attempted. The CMLPS-N1 cell line was successfully established from a sample obtained from a 16-year-old female Chinese rural dog with a diagnosis of liposarcoma.

### 2.2. Establishment and purification of CMLPS-N1 cell line

The tumor tissues were washed by PBS supplemented with 5% penicillin-streptomycin-amphotericin solution (Gibco, Billings, MA, USA) for three times and minced into 1 mm^3^ pieces. The blood, fat, and fibro connective tissues were removed during the washing procedure. The small tumor pieces were then incubated with collagenase type III (Gibco, Billings, MA, USA) and neutral protease (Gibco, Billings, MA, USA) at 37 °C in a humidified atmosphere of 5% CO_2_ for 3 h with continuous agitation. The digested tissue pieces were filtered through nylon mesh cloth (100 μm) and centrifuged at 1000 rpm for 5 min, and the pelleted cells were resuspended in DMEM (Gibco, Life Technologies) containing 15% FBS (Hycezmbio, Wuhan, China) and 1% penicillin-streptomycin solution (Gibco, Billings, MA, USA). The cell suspension was then transferred into a 25 cm^2^ cell culture flask and maintained at 37 °C in a humidified atmosphere of 5% CO_2_.

After forming a complete monolayer in the primary culture, cells were passaged to a new flask with fresh DMEM-15% FBS medium. When the following cell cultures were 80–90% confluent, cells were subjected to passage procedures: cells were washed with PBS, treated with 0.25% trypsin solution (Gibco, Billings, MA, USA), incubated until they dislodged from the flask surface, and split at a ratio of 1:2 in fresh DMEM-15% FBS medium. The cells were sampled and frozen every two passages. The cell line was subcultured continuously for 50 passages, and upon completion of the 50th passage, the established cell line was designated as CMLPS-N1 cells. The CMLPS-N1 cells were negative for Mycoplasma (Supplementary Figure 1).

### 2.3. Cell culture

The MCF-7 human breast cancer cell line was procured from the American Type Culture Collection (ATCC, Manassas, VA, USA). The CHMp and CMT-7364 canine mammary tumor cell lines were generously supplied by Professor Degui Lin from China Agricultural University (Beijing, China). All cell lines were cultured in DMEM (Gibco, Billings, MA, USA) supplemented with 10% FBS (Hycezmbio, Wuhan, China) and 100 U/mL of penicillin-streptomycin (Gibco, Billings, MA, USA) at 37 °C in a humidified atmosphere of 5% CO_2_.

### 2.4. Oil Red O staining

Cells were seeded in 24-well plates at a density of 1 × 10^4^ cells per well and cultured to 50% confluence. The medium was removed, and cells were washed with PBS twice. Each well was added with 200 μL of 4% paraformaldehyde and cells were fixed at room temperature (RT) for 15 min. After being washed with PBS, cells were treated with 60% isopropanol for 2 min and stained with 0.5% Oil Red O for 15 min, followed by washing with 60% isopropanol and PBS. Digital images were captured using an optical microscope (Olympus, BX53, Tokyo, Japan).

### 2.5. Electron microscopy

Cells at passage 30 were processed for transmission electron microscopy. Cells were harvested from 6-well plates after reaching 80–90% confluence. The medium was discarded, and the cells were fixed with 2.5% glutaraldehyde electron microscopy fixative at RT for 5 min. Cells were gently scraped off with a cell scraper and collected into centrifuge tubes. New fixative was added after centrifugation, and the suspended cells were transferred to 4 °C. The fixative was aspirated, and the cells were fixed with pre-chilled 1% osmium tetroxide in 0.1 M phosphate buffer (pH 7.4) at RT for 2 h. The samples were dehydrated through a graded ethanol series (50%, 70%, 80%, 90%, 95%, 100%) and permeabilized overnight with a 1:1 mixture of acetone and Epox812 embedding agent. Infiltration was performed with pure Epox812 resin overnight. Finally, embedding sections were stained with uranium-lead and observed under an FEI Tecnai G2 Spirit transmission electron microscopy. PBS was used for all washing steps.

### 2.6. Histological and immunohistochemistry analyses

Tissues were fixed in 4% formaldehyde solution, paraffin-embedded, and sections were stained with hematoxylin and eosin (H&E). IHC detection using antibodies specific for MDM2, Vimentin, PCK, Ki67, ER, PR, and HER-2 was performed on paraffin sections. In this detection, those without primary antibodies were negative controls. Cross-reactivity of primary antibodies with canine tissues involved in IHC and IF has been previously demonstrated. The staining processes were performed according to standard methods. The details of the primary antibodies used are provided in Supplementary Table S1.

### 2.7. Karyotype analysis

For karyotype analysis, cells in the logarithmic growth phase were treated with 0.05 mg/mL colchicine for 5 h. Then, adherent cells were dissociated with 0.25% trypsin and resuspended in 0.075 M hypotonic KCl (preheated to 37 °C) for 30 min at 37 °C. The cell suspension was then fixed in methanol: acetic acid (3:1) and subsequently dropped onto clean, pre-chilled glass slides. Finally, the prepared slides were stained with Giemsa for 15 min, and the chromosome numbers of the cells were counted in 50 metaphase cells. The chromosomes were observed and counted under an optical microscope.

### 2.8. Cell proliferation assay

Cells were distributed in 96-well plates at a density of 1 × 10^3^ cells per well and each well was inoculated with 100 μl of cell suspension and incubated at 37 °C in a 5% CO_2_ incubator. Cell viability was assessed every 24 h using the Cell Counting Kit-8 (CCK-8; Hycezmbio, Wuhan, China) following the manufacturer’s instructions. The absorbance at 450 nm was quantified using a multifunctional microplate reader (BMG LRBTECH, SPECTORstar, Offenburg, Germany). The cell growth curve was established using the standard curve of the cell assay with a known OD value, and the doubling time was calculated based on the regression equation of the curve using GraphPad Prism 9 software.

### 2.9. Colony formation assay

Cells were seeded in 6-well plates at a density of 200 cells per well and then gently shaken in a cross direction to disperse the cells evenly. The 6-well plates were transferred to a 37 °C incubator and cells were cultured for 2–3 weeks with medium being replaced every three days. When clones became visible, the culture samples were harvested. The cells were washed with PBS twice, fixed with methanol for 20 min, stained with 0.1% crystalline violet staining solution for 30 min, and finally washed by running water. After that, the number of clones with more than 50 cells was counted under a microscope, and the cloning efficiency of cells was calculated according to the formula: Clone formation rate (%) = (number of clones/number of inoculated cells) × 100%.

### 2.10. Wound healing assay

Cells were cultured in 6-well plates to produce monolayers and reached 80% confluence. Wounds were generated using a 200 μL pipette tip, which was employed to vertically scrape the cell layer in places where cells were densely packed. Subsequently, the cells were gently washed with PBS twice to eliminate any detached cells. Following this, the cells were replenished with Opti-MEM I media (Gibco, Billings, MA, USA) that had been decreased in serum content. Photographs of the wounds were taken at the time of the initial introduction of the scrape wound and again after a 24 h period using an inverted microscope (Olympus, CKX41, Tokyo, Japan). The distances of the optical wound were measured utilizing the ImageJ program.

### 2.11. Transwell assay

To detect cell invasion ability, firstly, matrigel (Biozellen, Ord, NE, USA) was diluted 18-fold before smoothly spreading on the upper chamber of a 24-well transwell insert (Biofil, Guangzhou, China). After the matrigel became solidified at 4 °C, cells were seeded into the matrigel-coated chambers at a density of 6 × 10^4^ cells per chamber. Meanwhile, a 500 μL complete medium was added to the lower chamber of the transwell inserts. After incubation for 24 h, the non-invaded cells on the upper side of the upper chamber were removed by scraping with a cotton swab, while the invaded cells on the lower side of the upper chamber were fixed with methanol and then stained with crystal violet. An optical microscope was utilized to capture images.

### 2.12. Immunofluorescence staining

Cells were seeded into 24-well plates at a density of 1 × 10^4^ cells per well and reached 50–60% confluence. Following this, the cells underwent fixation with 4% paraformaldehyde for 20 min, permeabilization with 0.2% Triton X-100 for 10 min, and blocking with 5% BSA for 2 h, and were incubated with the primary antibody at 4 °C overnight. The information about the primary antibodies is provided in Supplementary Table S1. Alexa Flour 594-Goat Anti-Rabbit IgG was used as a fluorescent secondary antibody and incubated with cells for 2 h. Finally, nuclei were stained with DAPI for 10 min. Image acquisition was performed using a fluorescence microscope (Olympus, IX73, Tokyo, Japan).

### 2.13. Western blot

Cells were seeded in 6-well plates. Upon reaching 80%–90% confluence, they were lysed using ice-cold RIPA buffer. The total protein concentration was measured with a bicinchoninic acid (BCA) protein quantification kit (Hycezmbio, Wuhan, China) to normalize the protein loading amount to 15 μg per lane. The proteins were separated by sodium dodecyl sulfate-polyacrylamide gel electrophoresis (SDS-PAGE) and transferred onto polyvinylidene difluoride (PVDF) membranes. The membranes were then blocked with 5% skim milk in TBST for 2 h at RT, followed by incubation with primary antibodies overnight at 4 °C. After washing, the membranes were incubated with horseradish peroxidase (HRP)-conjugated secondary antibodies for 2 h at RT. Finally, the signals were detected using a Fusion Solo S imaging system (Vilber, Paris, France) and analyzed with ImageJ software. The details of the primary antibodies used are provided in Supplementary Table S1.

### 2.14. Tumorigenicity assay

This study was approved by the Institutional Ethical Committee for Animal Care and Use of Huazhong Agricultural University (permit number: HZAUMO-2025-0028). All the experimental procedures adhered to the United States National Institutes of Health guidelines. Five-week-old Balb/C and Balb/C-nude female mice were purchased from the Experimental Animal Center of Huazhong Agricultural University (Wuhan, China) and maintained in standard conditions for one-week adaptation. The mice were used to investigate the tumorigenicity of the CMLPS-N1 cell line at passage 30. Approximately 3 × 10^6^ cells(Balb/C-nude) and 6 × 10^6^ cells(Balb/C) CMLPS-N1 cells were harvested and suspended in 100 μL of PBS, then subcutaneously injected into the mouse’s fourth mammary pad (*n* = 3). The volume of tumor was measured every 3 days and assessed using the following formula: V (mm^3^) = length × width^2^/2. After 36 days of tumor plantation, all mice were euthanized, and the xenograft tumors were meti­culously excised before being partly immersed with 4% paraformaldehyde for histological and IHC examinations.

### 2.15. Assessment of cytotoxic effects of chemotherapy drugs on CMLPS-N1 and CMT-7364 cells

To detect the inhibitory effect of Cisplatin, Paclitaxel, Epirubicin, and 5-Fluorouracil on CMLPS-N1 and CMT-7364 cells, cell viability was measured using Cell Counting Kit-8 as referred to in the previous part. Cells were distributed in 96-well plates with a density of 1 × 10^4^ cells per well, and upon reaching 50–60% confluence, they were treated with different concentrations of drugs for 24 h, 48 h, or 72 h. The previous medium mixed with drugs was then replaced by CCK-8 solution. After incubation for 40 min, the absorbance at 450 nm was quantified using a multifunctional microplate reader.

### 2.16. Myoplasma detection

To confirm the cells were not contaminated with mycoplasma, the Mycoplasma Test Kit (Beyotime, Shanghai, China) was utilized according to the manufacturer’s instructions. Briefly, cells were planted in 6-well plate at a density of 1 × 10^4^ cells per well, with mycoplasma-free cells as a negative control. After 5 days of cell culture, the medium was replaced with 1 mL of fixative. Following fixation and air-drying, cells were stained with 1 mL of Hoechst 33258 working solution at 37 °C for 15 min in the dark. The solution was removed, and cells were washed three times with 2 mL of sterile ultrapure water. Fluorescence microscopy under UV excitation was used to identify blue fluorescent dots or bead-like particles around the cells.

### 2.17. Statistical analyses

Data are presented as the mean ± standard deviation (mean ± SD) of at least three independent ex­­periments. A one-way analysis of variance (ANOVA) followed by Tukey’s post hoc test was used for an analysis with multiple comparisons. A value of *p < 0.05 was considered statistically significant. Values of **p < 0.01, ***p < 0.001, or ****p < 0.0001 were considered highly significant, and *p* > 0.05 (ns) was considered not statistically significant. All analyses were performed using GraphPad Prism 9.0

## 3. Results

### 3.1. Establishment of the CMLPS-N1 cell line from a canine mammary liposarcoma

**Figure 1.**
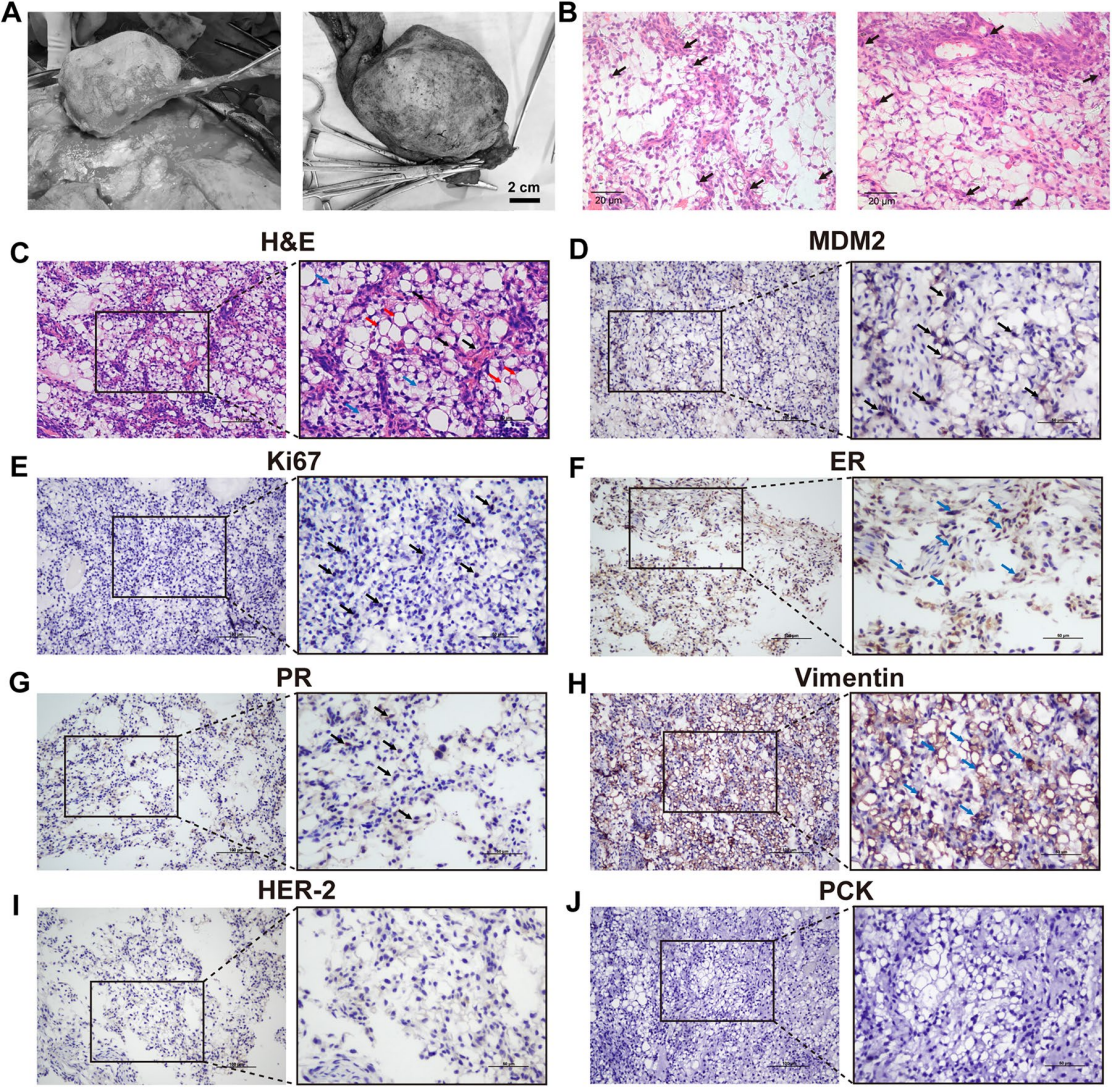
Histopathological and immunohistochemical profile of the CMLPS-N1 parental tumor. (A) Intraoperative photograph of mastectomy for CMLPS-N1 primary tumor (left), and the resected CMLPS-N1 parental tumor (right). Scale bar: 2 cm. (B) H&E-stained section of the CMLPS-N1 parental tumor showing increased mitotic figures (arrows). Scale bar: 20 μm. (C) H&E-stained section of the CMLPS-N1 parental tumor showing adipocytes of varying sizes arranged in sheets (red arrows), increased stromal fibroblasts (black arrows), and partial stromal myxoid degeneration (bule arrows). (D-J) IHC stain of the CMLPS-N1 parental tumor. Scale bar: 100 μm (left), 50 μm (right). (D) MDM2 protein expression was positive, with immunoreactivity observed in both the nucleus and cytoplasm (black arrows). (E) Positive Ki67 immunoreactivity was strictly confined to the nucleus (black arrows). (F) ER positivity was detected in both the cytoplasmic and nuclear compartments (blue arrows). (G) PR immunoreactivity demonstrated predominant nuclear localization (black arrows). (H) Strong Vimentin immunoreactivity was observed in both the nucleus and cytoplasm (blue arrows). (I) HER-2 protein expression was negative. (J) PCK expression was undetectable in the tissue.

Cases of canine mammary tumors were collected from Huazhong Agricultural University Veterinary Teaching Hospital. We initiated 30 primary cultures from CMT tissues obtained from female dogs. From these cultures, a novel cell line, designated CMLPS-N1, was successfully isolated, purified, and maintained for over 80 passages in our laboratory. This indicates its long-term stability and potential for further research. CMLPS-N1 was derived from a mammary tumor in a 16-year-old female dog. The tumor, located near the right fourth mammary gland, exhibited rapid growth and was surgically excised *via* mastectomy (Figure 1A). The tumor recurred within six months post-surgery, led to systemic metastasis, and ultimately resulted in the dog’s death.

The resected tumor tissue was firm and gelatinous. It was encapsulated and exhibited extensive involvement with noticeable hemorrhage (Figure 1A). Histopathological analysis revealed adipocytes of varying sizes arranged in sheets, with mild nuclear atypia. There were increased stromal fibroblasts and partial myxoid degeneration of the stroma (Figure 1C). Under high magnification, the mitotic count was confirmed to be 6 per high-power field (Figure 1B). Notably, the cellular pleomorphism and elevated mitotic activity of these features collectively indicate clear malignant characteristics. Immunohistochemical analysis revealed positive expression of MDM2 (Figure 1D), a liposarcoma marker, along with Vimentin, ER, PR, and Ki67 (Figure 1E-H), while HER-2 and PCK were negative (Figure 1I,J). These results align with the histopathological features of canine mammary liposarcoma.

### 3.2. Microscopic morphology of CMLPS-N1 cells

**Figure 2.**
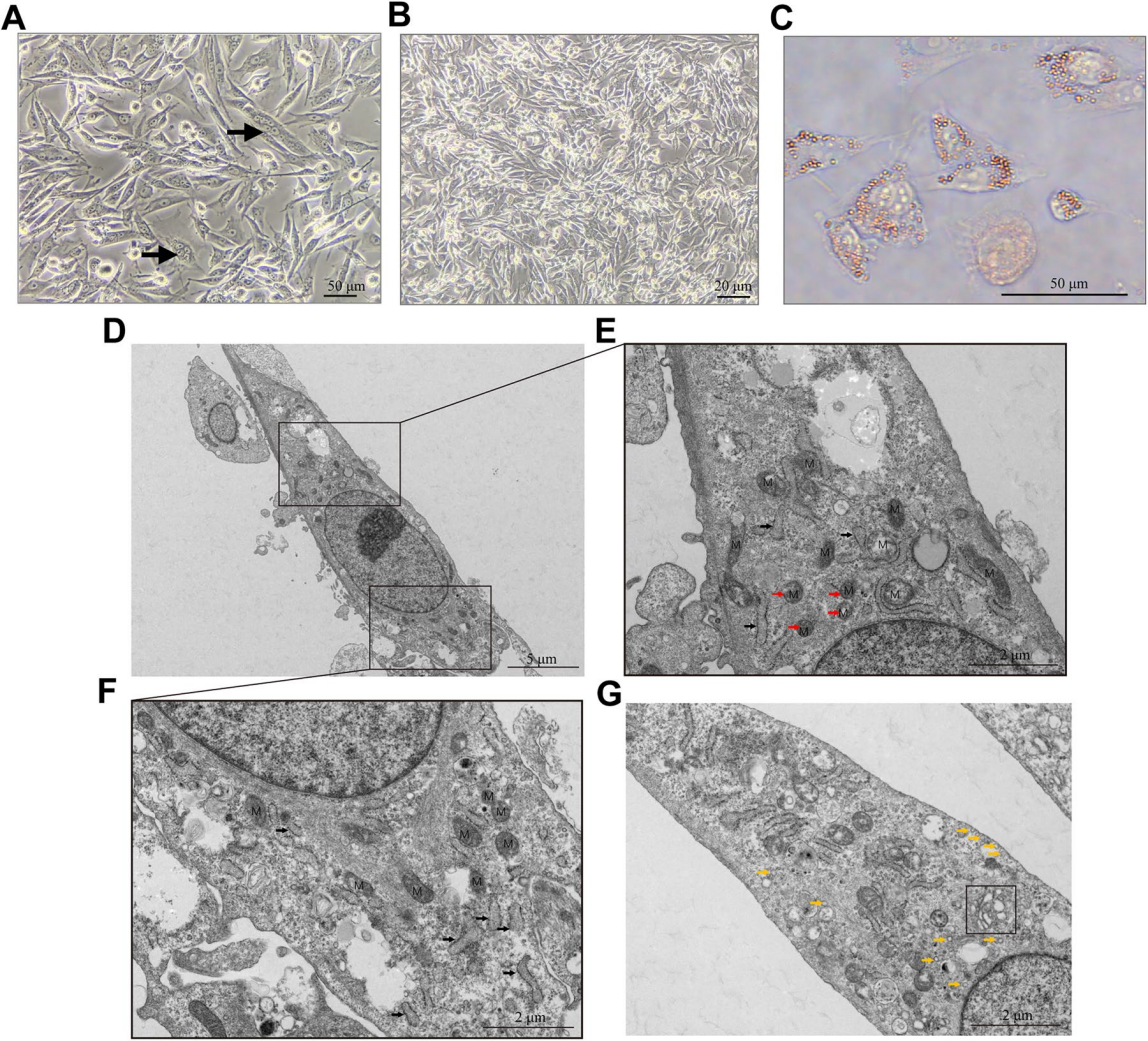
Microscopic morphology of CMLPS-N1 cells. (A) Cells exhibited a spindle-to-polygonal morphology and were arranged in a fibroblast-like pattern under microscopic observation. Multinucleated tumor giant cells were observed (arrows). Scale bar: 50 μm. (B) At low magnification, the cells at full confluence were observed to form overlapping multi-layered structures. Scale bar: 20 μm. (C) Oil Red O staining was used to visualize lipid droplets within the cells. Scale bar: 50 μm. (D-G) Transmission electron micrograph of cells at passage 30. The cytoplasm contained numerous mitochondria (labeled with ‘M’), including immature forms (red arrows), abundant rough endoplasmic reticulum, with focal dilation (black arrows), and scattered vacuoles (yellow arrows). Golgi apparatus was also present (black square). Scale bar: 5 μm (D), 2 μm (E-G).

Under microscopic observation, the cells exhibited a spindle-to-polygonal morphology and were arranged in a fibroblast-like pattern. Loss of contact inhibition was observed during cell proliferation, accompanied by the presence of multinucleated tumor giant cells (Figure 2A). At full confluence, the cells demonstrated the capacity to form overlapping layers (Figure 2B). Together, these features are characteristic of malignant cells *in vitro*. The result of Oil Red O staining revealed that over 95% of the cells contained cytoplasmic lipid droplets, which appeared as densely clustered orange-stained structures (Figure 2C).

Transmission electron microscopy further characterized the cells as elongated and spindle-shaped, with large nuclei, prominent nucleoli, and a high nuclear-to-cytoplasmic ratio (Figure 2D). The cytoplasm was rich in mitochondria. Some mitochondria displayed intact cristae and well-defined double membranes, while others exhibited indistinct cristae and outer membranes (Figure 2E,F). Immature mitochondria were also observed (red arrows) (Figure 2E). The cytoplasm contained abundant rough endoplasmic reticulum, with some regions showing dilation (black arrows) (Figure 2E,F). Golgi apparatus and lysosomes were also identified (Figure 2G). Additionally, numerous vacuoles were scattered throughout the cytoplasm (yellow arrows) (Figure 2G).

### 3.3. CMLPS-N1 is hypodiploid with a broad range of chromosomal numerical abnormalities

**Figure 3.**
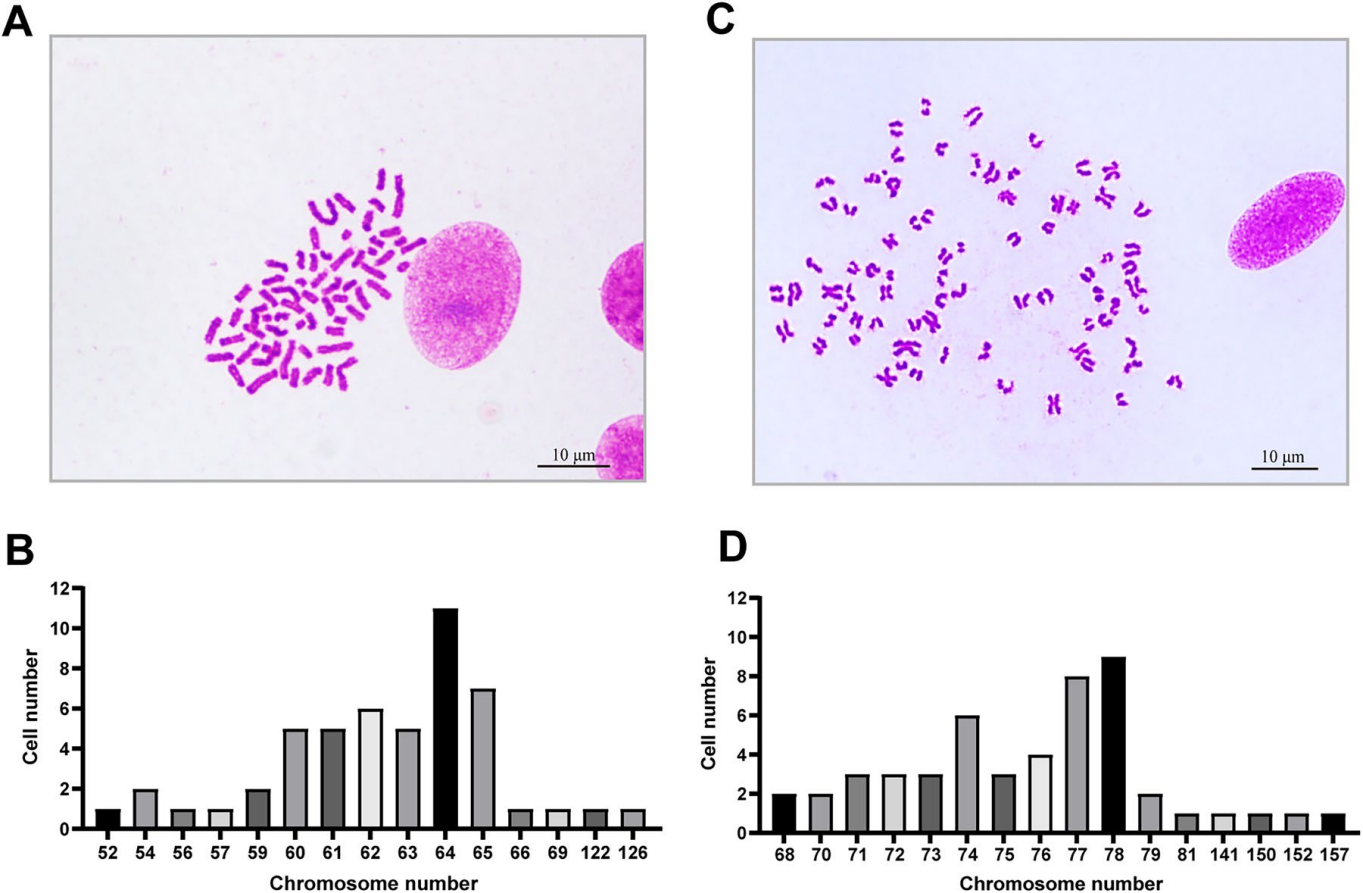
Karyotype analysis of CMLPS-N1 cells. (A,B) Karyotype analysis showed that the chromosome number of CMLPS-N1 cells cells ranged from 52 to 126, with a median of 63. Scale bar: 10 μm. (C,D) Karyotype analysis confirmed a normal diploid chromosome number (2n = 78) in canine fibroblasts. Scale bar: 10 μm.

Karyotype analysis was performed on 50 metaphase cells with well-dispersed, non-overlapping, and intact chromosomes under microscopy. Canine fibroblasts, which have a normal diploid chromosome number of 78, were used as a reference (Figure 3C,D). However, the CMLPS-N1 cell line exhibited numerical chromosomal abnormalities (Figures 3A,B). The chromosome number in these cells ranged from 52 to 126, with a median of 63, indicating hypodiploidy compared to the normal canine somatic cell karyotype.

### 3.4. Proliferation and motility of CMLPS-N1 cells *in vitro*

**Figure 4.**
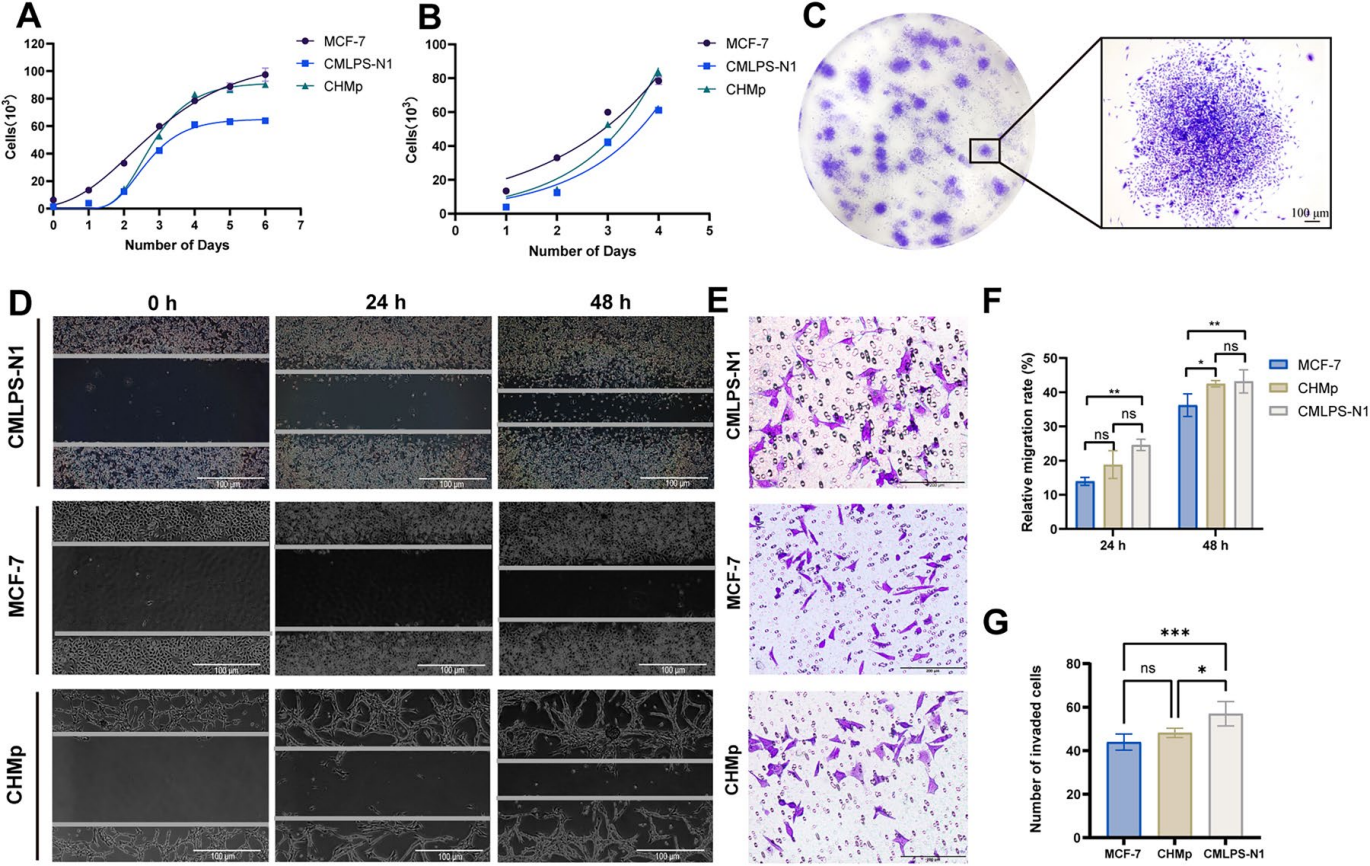
Proliferation and motility of CMLPS-N1 cells *in vitro*. (A) Growth curve analysis showed the cell proliferation ability of CMLPS-N1, compared to that of the human breast cancer cell line MCF-7 and the canine breast cancer cell line CHMp. (B) The logarithmic growth phase of these three cell lines. (C) Colony formation assays showed that CMLPS-N1 cells were capable of forming colonies at low seeding densities. Scale bar: 100 μm. (D,F) Wound healing assay was performed to evaluate the migration capacity of CMLPS-N1, MCF-7, and CHMp cells. Scale bar: 100 μm. (E,G) Transwell assay of these three cell lines to measure the cell invasion ability. Scale bar: 200 μm. Data are presented as the mean ± SD from five independent experiments (*n* = 5). *p < 0.05, **p < 0.01, ***p < 0.001, ns (not significant; *p* ≥ 0.05).

The cell proliferation ability of CMLPS-N1 was assessed and compared to that of the human breast cancer cell line MCF-7 and the canine breast cancer cell line CHMp. Growth curve analysis revealed a typical sigmoidal pattern for all three lines (Figure 4A). The doubling times, calculated using data from days 1 to 4 of the logarithmic growth phase, were 25.4, 36.2, and 23.3 h for CMLPS-N1, MCF-7, and CHMp, respectively (Figure 4B). Furthermore, colony formation assays demonstrated that CMLPS-N1 cells were capable of forming colonies even at low seeding densities, with a colony formation rate exceeding 20% (Figure 4C). This indicates that CMLPS-N1 cells exhibited robust self-renewal and proliferative potential, allowing them to bypass contact inhibition and thrive even at low cell densities.

Cell migration and invasion abilities were assessed through wound healing and transwell invasion assays. In the wound healing assay, CMLPS-N1 cells demonstrated higher migration capacity than both MCF-7 and CHMp cells, with wound closure rates of 24.6% and 43.2% at 24 and 48 h, respectively (Figure 4D,F). Consistent with this, the transwell invasion assay revealed that CMLPS-N1 cells exhibited higher invasiveness, with 57 cells invading through the membrane, compared to 44 for MCF-7 and 48 for CHMp (Figure 4E,G). These results collectively demonstrate that the CMLPS-N1 cell line possesses strong migratory and invasive capabilities.

### 3.5. Immunofluorescence analysis of molecular markers in CMLPS-N1 cells

**Figure 5.**
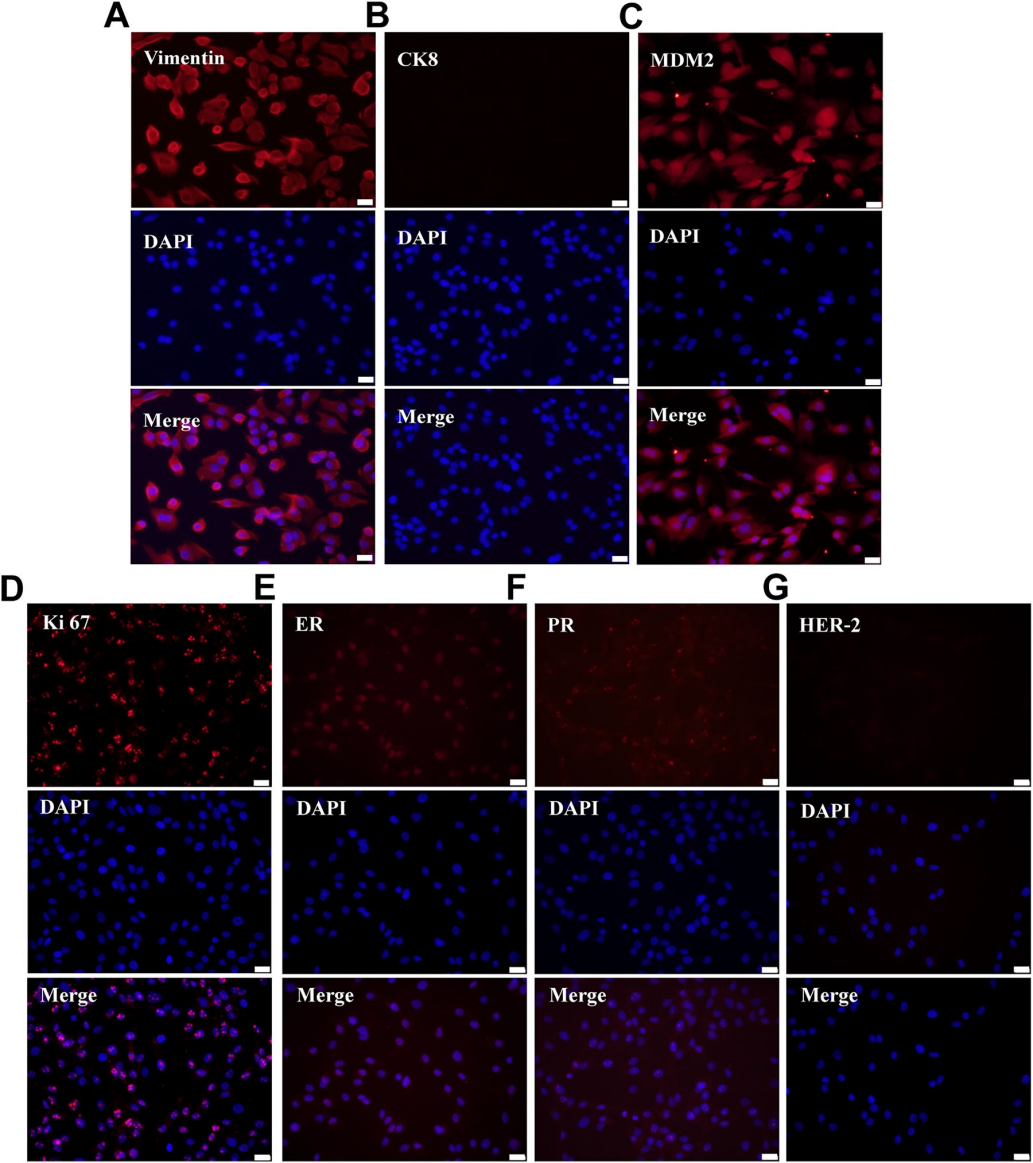
Immunofluorescence analysis of molecular markers in CMLPS-N1 cells. (A) Vimentin showed robust signals in nuclear and cytoplasmic compartments. (B) CK8 expression was undetectable in CMLPS-N1 cells. (C) MDM2 protein expression was positive, observed in both the nucleus and cytoplasm. (D) Ki67 immunofluorescence was exclusively nuclear. (E) ER exhibited stronger nuclear staining compared to cytoplasmic signals. (F) PR demonstrated predominant nuclear localization. (G) HER-2 showed complete negativity. Scale bar: 50 μm.

The expression of MDM2, Vimentin, CK8, Ki67, ER, PR, and HER-2 in CMLPS-N1 cells was detected by immunofluorescence (IF). In this experiment, the negative control without a primary antibody was used. The results showed that CK8 and HER-2 were negative (Figure 5B,G), while Vimentin (Figure 5A), MDM2 (Figure 5C), Ki67 (Figure 5D), ER (Figure 5E), and PR (Figure 5F) wer positive. The IF staining pattern of CMLPS-N1 cells was consistent with the immunohistochemical staining results of the parental canine mammary liposarcoma from which it was derived. These findings indicate that the cell line retained the molecular phenotype of the original tumor cells.

### 3.6. Comprehensive molecular profiling of CMLPS-N1 reveals its distinct identity

**Figure 6.**
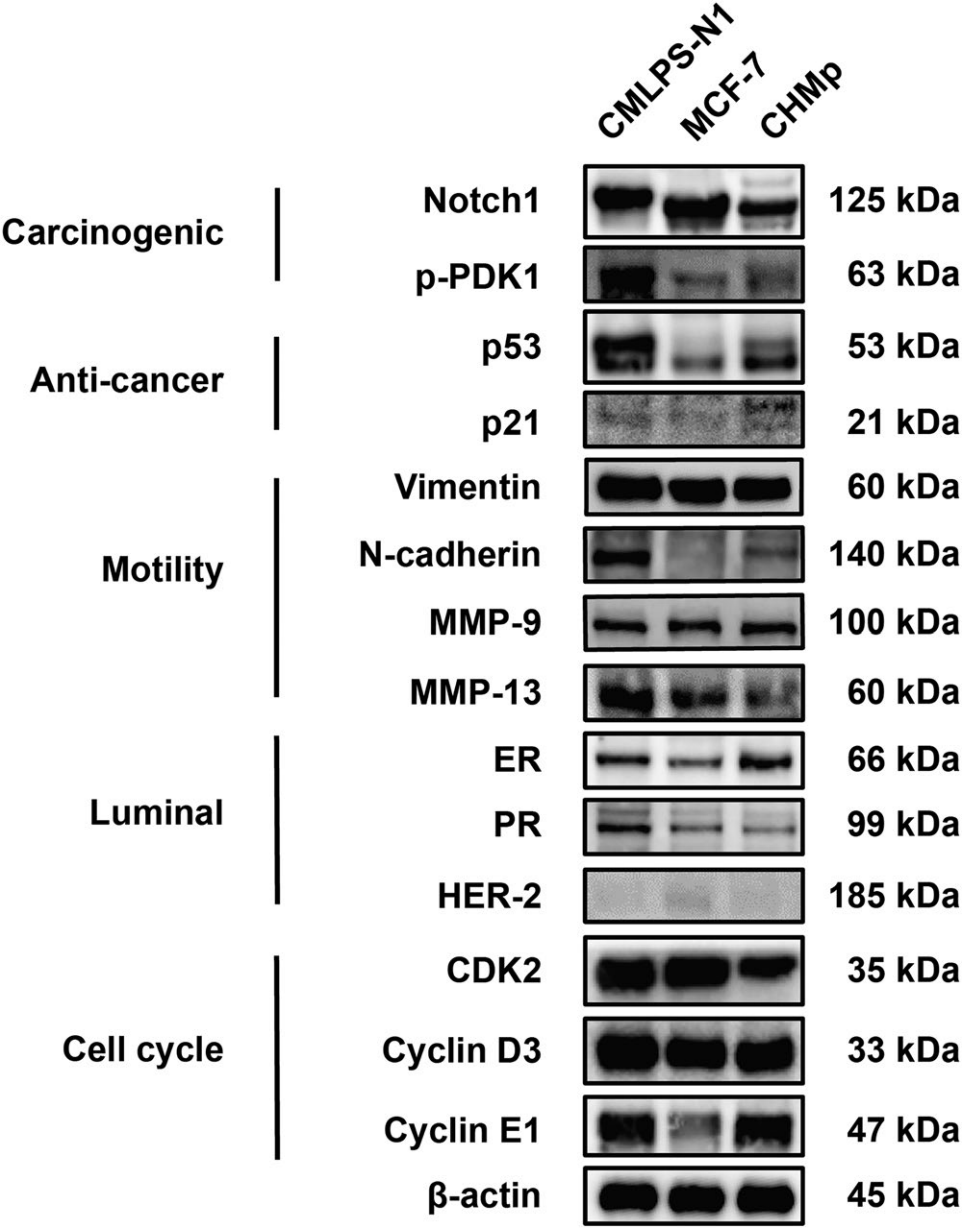
Comprehensive molecular profiling of CMLPS-N1 reveals its distinct identity. Western blot analysis was performed to compare the protein expression patterns of the CMLPS-N1 cell line with those of the human breast cancer cell line MCF-7 and the canine cell line CHMp. High expression levels of oncogenic proteins (Notch1, p-PDK1), cell cycle regulators (CDK2, Cyclin D3, Cyclin E1), and p53. The cells were positive for motility markers (Vimentin, N-cadherin, MMP-9, MMP-13) and hormone receptors (ER, PR), but negative for HER-2.

To molecularly characterize the newly established CMLPS-N1 cell line and define its oncogenic phenotype, we performed a comprehensive western blot analysis and compared its profile to the human breast cancer cell line MCF-7 and the canine breast cancer cell line CHMp (Figure 6). CMLPS-N1 exhibited high-level expression of the oncogenic proteins Notch1 and phosphorylated PDK1. Notably, unlike the MCF-7 cell line, which is a p53 wild-type model where the protein is rapidly ubiquitinated and degraded, resulting in low detectable levels, the CMLPS-N1 line showed exceptionally high levels of p53 protein, suggesting it harbors a stabilizing mutation that is characteristic of aggressive cancers with enhanced invasion, metastasis, and proliferation. Consistent with dysfunctional p53 pathway signaling, expression of its downstream target p21 was low. Furthermore, the cells displayed a highly motile and invasive phenotype, evidenced by high expression of the motility-associated markers Vimentin and N-cadherin, which was complemented by elevated levels of matrix metalloproteinases (MMPs), key enzymes facilitating extracellular matrix degradation and invasion. The hormone receptors ER and PR were detected, while HER-2 was negative; which were consistent with IF and IHC results. Finally, the high proliferative capacity of this cell line was underscored by the robust expression of cell cycle regulators CDK2, Cyclin D3, and Cyclin E1. Collectively, this integrated protein expression profile defines the CMLPS-N1 cell line as a novel and aggressive breast cancer cell model with a dysfunctional p53 pathway, a pro-metastatic motility signature, and high cyclin-driven proliferative potential.

### 3.7. Tumorigenesis potentials of CMLPS-N1 cells in mice

**Figure 7.**
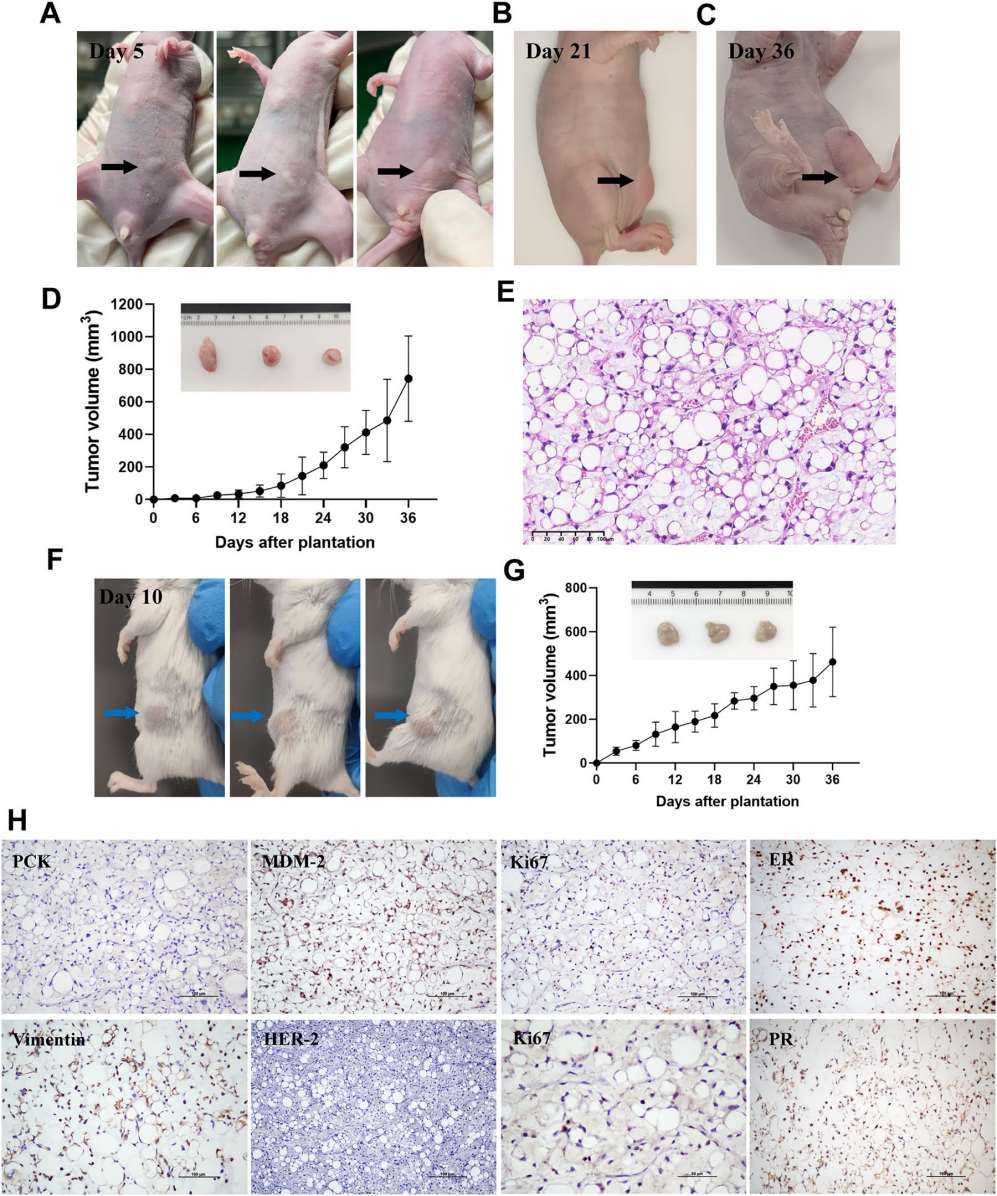
Tumorigenesis potentials of CMLPS-N1 cells in mice. (A) Tumor growth in BALB/c-nude mouse xenograft models implanted with CMLPS-N1 cells at day 5 post-inoculation. (B,C) Xenograft tumor progression at day 21 (B) and day 36 (C) after implantation of CMLPS-N1 cells in nude mice. (D) Tumor volume growth curve and excised xenograft photographs of nude mouse transplantable tumors. (E) H&E staining of the CMLPS-N1 nude mouse transplantable tumors. Scale bar: 100 μm. (F) Tumor growth in BALB/c mouse xenograft models implanted with CMLPS-N1 cells at day 10 post-inoculation (G) Tumor volume growth curve and excised xenograft photographs of BALB/c mouse transplantable tumors. (H) Immunohistochemical analysis of CMLPS-N1 xenograft tumors revealed negative staining for PCK and HER-2, but positive expression of MDM2, Vimentin, ER, PR, and Ki-67. Scale bar: 100 μm.

The tumorigenic potential of CMLPS-N1 cells was evaluated in both BALB/c and BALB/c-nude mice. In BALB/c-nude mice, visible white protrusions were observed at the injection sites in all three mice 5 days post-inoculation (Figure 7A). By day 21, tumors with maximum diameters ranging from 7 to 11 mm had developed, resulting in a 100% tumor formation rate (Figure 7B). At day 36, the mice were euthanized, and the tumors were excised (Figure 7C). The tumor volume growth curve exhibited an increasing growth rate over time, with the final volume reaching 700 mm³ (Figure 7D). Histological analysis of the implanted tumor tissue was performed using H&E staining. As shown in Figure 7E, the tumor tissue exhibited prominent vasculature, along with adipocytes of varying sizes distributed throughout the tissue. Additionally, frequent mitotic figures were observed. These findings are consistent with the characteristic features of liposarcoma. In BALB/c mice, visible tumor protrusions developed at the injection sites in all three mice 10 days post-inoculation (Figure 7F). By day 36, tumors with maximum diameters ranging from 8 to 12 mm had formed (Figure 7G), also yielding a 100% tumor formation rate.

Immunohistochemical staining of the mouse mammary liposarcoma tissues revealed that PCK and HER-2 were negative, while MDM2, Vimentin, ER, PR, and Ki67 were positive (Figure 7H). These results were consistent with the IF findings in the CMLPS-N1 cells. Together, these data confirmed that the xenograft tumors derived from CMLPS-N1 cells retained the differentiation and molecular phenotypes of the original tumor cells, supporting their common origin.

### 3.8. CMLPS-N1 as a novel model for chemotherapy drug screening: comparative inhibition with CMT-7364

**Figure 8.**
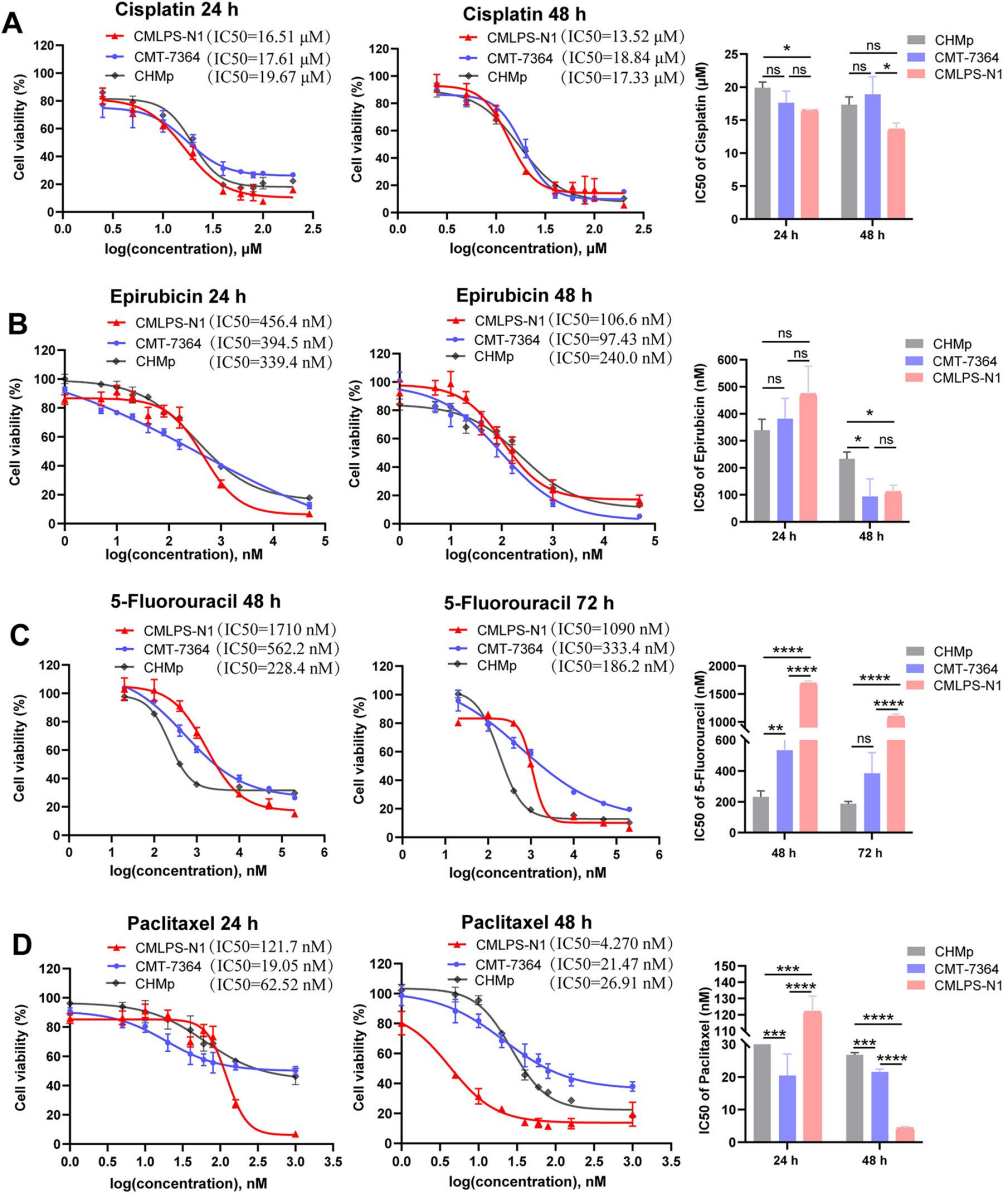
Cytotoxicity assays performed using cell counting kit-8 on CMLPS-N1 and CMT-7364 cell lines. (A) CMLPS-N1, CMT-7364 and CHMp cells were treated with increasing concentrations of cisplatin for 24 h and 48 h. The IC50 values are shown as follows. 24 h (CMLPS-N1: 16.51 μM; CMT-7364: 17.61 μM; CHMp: 19.67 μM) and 48 h (CMLPS-N1: 13.52 μM; CMT-7364: 18.84 μM; CHMp: 17.33 μM). (B) Epirubicin treatment was applied to these three cell lines at indicated doses for 24 and 48 h. IC50 values are shown as follows. 24 h (CMLPS-N1: 456.4 nM; CMT-7364: 394.5 nM; CHMp: 339.4 nM) and 48 h (CMLPS-N1: 106.6 nM; CMT-7364: 97.43 nM; CHMp: 240 nM). (C) CMLPS-N1 and CMT-7364 cells were treated with indicated concentrations of 5-fluorouracil for 48 h and 72 h. IC50 values are shown as follows. 48 h (CMLPS-N1: 1710 nM; CMT-7364: 562.2 nM; CHMp: 228.4 nM) and 72 h (CMLPS-N1: 1090 nM; CMT-7364: 333.4 nM; CHMp: 186.2 nM) (D) Paclitaxel sensitivity was determined after 24h and 48 h. IC50 values are shown as follows. 24 h (CMLPS-N1: 121.7 nM; CMT-7364: 19.05 nM; CHMp: 62.52 nM) and 48 h (CMLPS-N1: 4.27 nM; CMT-7364: 21.47 nM; CHMp: 26.91 nM). Data are presented as the mean ± SD from five independent experiments (*n* = 5). *p < 0.05, ***p < 0.001, ****p < 0.0001, ns (not significant; *p* ≥ 0.05).

To investigate the inhibitory effects of different chemotherapeutic agents on CMLPS-N1 cells, we selected four first-line anticancer drugs: cisplatin, epirubicin, 5-fluorouracil, and paclitaxel. CHMp and CMT-7364 cells were used as reference for comparison in cell viability assays. The results showed that cisplatin and epirubicin exhibited similar inhibitory effects on these three cell lines. After 48 h of treatment, the IC50 values for cisplatin were 13.52 µM for CMLPS-N1, 18.84 µM for CMT-7364 and 17.33 μM for CHMp (Figure 8A). For epirubicin, the IC50 values were 106.6 nM for CMLPS-N1, 97.43 nM for CMT-7364 and 240 μM for CHMp (Figure 8B). In contrast, 5-fluorouracil demonstrated significantly higher inhibitory efficacy against CMT-7364 (IC50: 526.2 nM at 48 h) and CHMp (IC50: 228.4 nM at 48 h) compared to CMLPS-N1 (IC50: 1710 nM at 48 h) (Figure 8C). Conversely, paclitaxel showed a stronger inhibitory effect on CMLPS-N1 (IC50: 4.27 nM at 48 h) than on CMT-7364 (IC50: 21.47 nM at 48 h) and CHMp (IC50: 26.91 nM at 48 h) (Figure 8D). These results indicate that the CMLPS-N1 cell line is suitable for evaluating the inhibitory effects of anticancer drugs on tumor cells, and represents a valuable cellular model for the development of chemotherapeutic agents targeting canine mammary sarcoma.

## 4. Discussion

Canine mammary tumor (CMT) is the most common type of tumor in intact female dogs, and its malignancy rate has been steadily increasing in recent years (Zheng et al. 2022; Varney et al. 2023). With the continued growth of the canine population, the health burden posed by CMT is also rising. Notably, naturally occurring CMT shares genetic, histopathological, and environmental similarities with human breast cancer (Kim et al. 2020; Kwon et al. 2023). These parallels have led to its widespread recognition as a valuable spontaneous animal model, offering important insights into the pathogenesis and therapeutic strategies of human breast cancer.

Canine mammary sarcoma (CMS) is a rare subtype of CMT, accounting for approximately 5.1% of all malignant cases (Dolka et al. 2013). However, its aggressive nature, high metastatic potential, and poor prognosis pose a significant health risk to affected dogs (Misdorp et al. 1971; Serin and Aydogan 2009). Moreover, CMT exhibits progressive malignant transformation from complex carcinoma to simple carcinoma and sarcoma (Hellmén et al. 1993). Given these characteristics, further research on CMS is crucial for improving the management and prognosis of dogs with CMT. This study reports the establishment of the first canine mammary liposarcoma cell line, designated CMLPS-N1, which has been stably passaged for over 80 generations. Derived from a spontaneously occurring canine mammary liposarcoma, CMLPS-N1 exhibits an abnormal karyotype, high proliferative and migratory capacity, along with strong tumorigenicity in mice. Molecular profiling showed positive expression of MDM2, Vimentin, p53, Ki67, ER, and PR, while PCK and HER-2 were negative. Additionally, cytotoxicity assays with chemotherapy drugs confirmed its potential as a model for drug screening.

Cell lines are fundamental tools in biomedical research, providing a controlled *in vitro* system to investigate cellular growth, differentiation, and metabolism. They also enable the modeling of disease progression and serve as platforms for evaluating drug safety and efficacy. Recent studies have demonstrated the diverse applications of CMT cell lines. Zhang et al. utilized CMT-1211 cells to show that miR-479 induces apoptosis *via* the IRAK2/NF-κB axis (Zhang T et al. 2021). Zhou et al. employed CMT-7364 as an *in vitro* model to assess the synergistic effects of the autophagy inhibitor HCQ and the glucose analog 2-DG in CMT treatment (Zhou N et al. 2022). Additionally, Bird et al. fused CMT-12, CMT-27, and CMT-28 cells with canine dendritic cells to develop a vaccine that enhances immune recognition of mammary tumors, contributing to advancement in CMT immunotherapy (Bird et al. 2011). Currently, several CMT cell lines have been established, including CMT-1 to CMT-6 (Wolfe et al. 1986), CHMp (Uyama et al. 2006), CMT-7364 (Zhang H et al. 2018), and CMT-U27 (HellmÉn 1992). However, most CMT research has focused on mammary carcinoma, and the majority of available cell lines are derived from epithelial tissue. In contrast, cell lines originating from mesenchymal tissue remain scarce, with only one reported case of a canine mammary osteosarcoma cell line from Japan (Kawabata et al. 2006). CMLPS-N1, derived from canine mammary liposarcoma, exhibits high proliferative and migratory capacity and demonstrates tumorigenicity in both BALB/c and BALB/c-nude mice. These findings fill a critical gap in the study of CMT, particularly CMS and liposarcoma subtypes. While this study provides initial *in vivo* evidence, the xenograft experiment was conducted with a limited cohort. Future investigations with larger animal numbers across diverse mouse models will be essential to fully delineate the tumorigenic potential and *in vivo* biology of CMLPS-N1.

MDM2 is a ubiquitin ligase that primarily functions as a negative regulator of the tumor suppressor gene p53 (Adams et al. 2023). It binds to the N-terminal transcriptional activation domain of p53, promoting its degradation, and also inhibits its transcriptional activity (Haupt et al. 1997). In human oncology, MDM2 gene amplification serves as a diagnostic marker for well-differentiated and dedifferentiated liposarcomas, and IHC detection of MDM2 is considered a surrogate marker for MDM2 gene amplification (Yoshimatsu et al. 2022). The immunohistochemical expression of MDM2 in canine liposarcomas has been reported, showing a pattern similar to that observed in humans. Specifically, positive immunohistochemical expression of MDM2 is significantly associated with well-differentiated and dedifferentiated canine liposarcomas (Avallone et al. 2016). Therefore, MDM2 IHC staining may serve as a valuable diagnostic marker for canine liposarcomas. In this study, MDM2 protein expression was detected in both primary tumor tissue and murine xenograft liposarcoma tissue, as well as in the CMLPS-N1 cell line. The protein was present in both the cytoplasm and nucleus. Additionally, the mesenchymal marker Vimentin showed strong positive expression, whereas the epithelial marker PCK was negative, further supporting the molecular characteristics of liposarcoma.

Ki67 expression is a critical marker for assessing cell proliferation and is commonly used to evaluate tumor malignancy (Carvalho et al. 2016; Tokuda et al. 2017). High Ki67 expression correlates with increased malignancy. Dolka et al. reported a significant association between Ki67 expression and the grading of canine mammary liposarcomas (Dolka et al. 2013). In this study, Ki67 was highly expressed in the nuclei of CMLPS-N1 cells, primary tumor tissues, and mouse xenograft tumors, indicating their malignant characteristics. Triple-negative breast cancer is a highly aggressive breast cancer subtype characterized by the absence of HER-2, PR, and ER expression and is associated with poor prognosis (Garrido-Castro et al. 2019). In our study, we assessed the expression of HER-2, PR, and ER in CMLPS-N1 cells. ER and PR expression was positive, consistent with previous findings in a canine metastatic mammary sarcoma (Polinas et al. 2016), whereas HER-2 expression was negative. These results indicate that the CMLPS-N1 cell line does not exhibit triple-negative breast cancer molecular characteristics, suggesting its potential as a model for investigating hormone therapy in CMT.

CMT is characterized by high heterogeneity, multifocal involvement, and diverse histological subtypes, which presents significant challenges for clinical treatment (Valdivia et al. 2021). Surgical resection is ineffective for canine inflammatory mammary carcinoma and often fails to prevent recurrence in metastatic cases (Stratmann et al. 2008). Consequently, the development of safe and effective chemotherapeutic agents for adjuvant therapy is essential to improve the prognosis of dogs with CMT. In human oncology, numerous chemotherapeutic drugs, such as alkylating agents, antimetabolites, microtubule inhibitors, and anthracycline antibiotics, have demonstrated substantial efficacy (Wang H et al. 2021; Wang B et al. 2022; Roskoski Jr 2024). However, the application of these human drugs in CMT treatment remains controversial (Zambrano-Estrada et al. 2018), highlighting the critical need for the development of canine-targeted chemotherapies. In this study, we investigated the inhibitory effects of four human chemotherapeutic drugs, cisplatin, epirubicin, 5-fluorouracil, and paclitaxel, on CMLPS-N1 cells, with CMT-7364 and CHMp cells as control. Our findings aim to assess the potential of CMLPS-N1 cells as a model for drug screening and pharmacological research.

CMLPS-N1 exhibited comparable sensitivity to cisplatin and epirubicin to that of CMT-7364 and CHMp. Notably, both drugs inhibit tumor growth by inducing DNA damage and triggering apoptosis (Shoji et al. 2022; Mallard et al. 2024), indicating that these three cell lines may have similar DNA damage repair efficiencies or comparable sensitivities of apoptotic pathways to DNA damage. Crespo et al. (2024) observed that epirubicin treatment of canine inflammatory mammary carcinoma cells (IPC-366) induced significantly stronger growth inhibition than observed in our study. However, their treatment duration was 72 h, substantially longer than the 24-hour exposure used in our study, which may account for the differences in drug sensitivity between the cell lines. As for the paclitaxel treatment, we found that a substantial proportion of CMT-7364 and CHMp cells remained viable under both short- and long-term paclitaxel treatment. In comparison, CMLPS-N1 displayed higher tolerance under short-term exposure but markedly reduced viability under prolonged treatment, irrespective of drug concentration. This difference may be attributed to enhanced G2/M checkpoint regulation in CMT-7364 and CHMp cells, potentially facilitating delayed or escaped cell cycle arrest. In contrast, CMLPS-N1 may achieve short-term tolerance through transient adaptive changes in microtubule dynamics. 5-Fluorouracil exerts its anticancer effects primarily through thymidylate synthase inhibition (Haritha et al. 2021). CMT-7364 and CHMp showed greater sensitivity to 5-fluorouracil than CMLPS-N1, possibly attributed to their lower thymidylate synthase expression. Additionally, variations in drug efflux activity may further explain the differences in drug tolerance between these cell lines (Nambaru et al. 2011; Stefanski et al. 2023). Together, these results support the potential of CMLPS-N1 as a valuable model for drug screening and pharmacological studies.

## 5. Conclusion

In conclusion, the present study established the first canine mammary liposarcoma cell line, which maintains stable biological functions with strong proliferative capacity, migratory ability, and tumorigenicity during prolonged passages. This cell line shows potential as a material for drug screening. The establishment of CMLPS-N1 enriches the CMT cell line repository and provides a valuable resource for CMS research. It is expected to play a significant role in future studies on the physiological functions, disease mechanisms, and translational applications of breast tumor cells.

## Supplementary Material

supplementary Figure1.tif

supplementary TableS1.xls

## Data Availability

All data generated or analyzed during this study are included in this published article.
