# Supplementary figures and images for: CMLPS-N1: a novel preclinical cell line model for canine mammary tumor and its application in therapeutic screening
Source: Vet Q. 2026 Jan 10;46(1):2614697. doi: 10.1080/01652176.2026.2614697 (PMC12794710; doi:10.1080/01652176.2026.2614697)

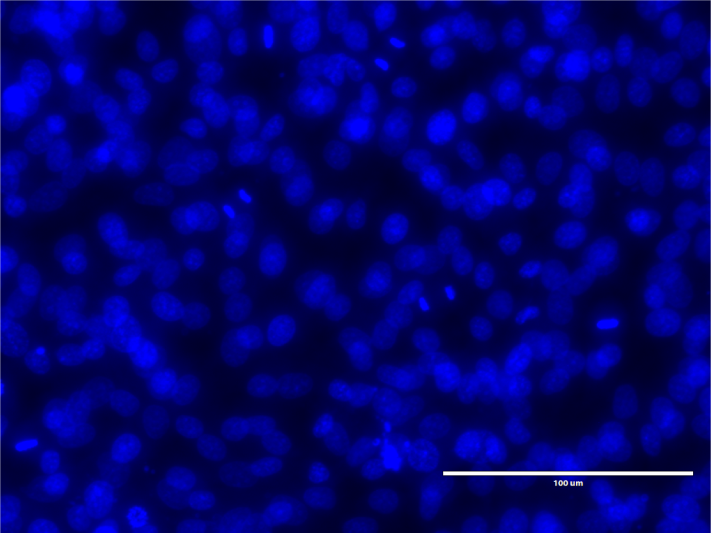

Supplement: supplementary Figure1.tif [file TVEQ_A_2614697_SM9800.tif]
